# Exploratory proteomic biomarker combinations associated with post-concussion symptoms following a concussion in adolescents

**DOI:** 10.3389/fneur.2026.1872460

**Published:** 2026-08-14

**Authors:** Jessica Gill, Arum Lim, Emily Dennis, Kevin Bickart, Sijung Yun, Joseph Yun, John Alice, Christopher Miles, Sara PD Chrisman, Christopher Vaughan, C. Munro Cullum, Lawrence J. Cook, Gerard Gioia, Frederick P. Rivara, Christopher C. Giza, Jeffrey J. Bazarian

**Affiliations:** 1 School of Nursing and School of Medicine, Department of Neurology, Johns Hopkins University, Baltimore, MD, United States; 2 School of Nursing, Johns Hopkins University, Baltimore, MD, United States; 3 Department of Neurology, University of Utah, Salt Lake City, UT, United States; 4 Department of Neurology, University of California, Los Angeles, Los Angeles, CA, United States; 5 Department of Family and Community Medicine, Wake Forest University School of Medicine, Winston-Salem, NC, United States; 6 Department of Pediatrics, University of Washington, and Seattle Children’s Research Institute, Seattle, WA, United States; 7 Pediatric Neuropsychologist, Division of Pediatric Neuropsychology, Children's National Hospital, Washington, DC, United States; 8 Department of Psychiatry, Neurology, and Neurological Surgery, University of Texas Southwestern Medical Center, Dallas, TX, United States; 9 Department of Pediatrics, University of Utah, Salt Lake City, UT, United States; 10 Department of Pediatrics and Psychiatry, George Washington University School of Medicine, Washington, DC, United States; 11 Children’s National Hospital, Washington, DC, United States; 12 Departments of Pediatrics and Neurosurgery, UCLA School of Medicine, University of California Los Angeles, Los Angeles, CA, United States; 13 Department of Emergency Medicine and Neurology, University of Rochester Medical Center, Rochester, NY, United States

**Keywords:** adolescents, biomarker, brain injury, concussion, inflammation, recovery

## Abstract

**Objective:**

This study examined the ability of established brain biomarkers, glial fibrillary acid protein (GFAP), neuro-filament light chain (NFL), ubiquitin carboxy hydrolase-L1(UCH-L1), tau, and phosphorylated tau (p-tau), and novel biomarkers from blood collected sub-acutely after concussion to determine prediction of persisting post-concussion symptoms (PPCS) beyond 3 months. We hypothesized that a combination of established and novel proteins would predict high PPCS burden.

**Participants:**

Adolescents 11 to 17.99 years with a concussion based on Concussion In Sport Group (CISG) criteria were eligible. Participants were assessed 7–35 days post-injury (baseline) and then reassessed at 3 months (follow-up) for persistent post-concussion symptoms (PPCS) using the Post-Concussion Symptom Inventory, 2nd Edition (PCSI-2). A total of 155 participants (78 females, 77 males) with both blood biomarkers and 3-month symptom data were analyzed.

**Design:**

Plasma proteins collected sub-acutely (7–35 days post concussion) were quantified by both Quanterix and the Olink Explore platform, and compared between participants with the highest and lowest quartiles of PPCS severity at follow-up (85–95 days post concussion) using the PCSI-2. An exhaustive best-subsets logistic regression strategy was executed following clinical and biological pre-filtering to identify parsimonious multivariable configurations of protein biomarkers distinguishing individuals with high and low PPCS at follow-up. A stratified 10-fold cross-validation framework was implemented to evaluate model generalizability and safeguard against overfitting, while bootstrapping was used to calculate confidence intervals. Ingenuity Pathway Analysis (IPA) was performed to generate hypotheses of molecular pathways implicated in PPCS pathogenesis.

**Results:**

None of the brain biomarkers collected sub-acutley were significantly different in PPCS-high and PPCS-low groups at 90-day follow-up. No Olink proteins survived multiple testing correction, but a cross-validated multivariable model, including Tripartite Motif Containing 39 (TRIM39) + Sclerostin (SOST) + Transcription factor Dp family member 3 (TFDP3) + TNF receptor superfamily member 9 (TNFRSF9) + Leptin (LEP) + Prune Homolog 2 With BCH Domain (PRUNE2) + Trimethylguanosine Synthase 1 (TGS1) + EPCAM (Epithelial Cell Adhesion Molecule) distinguished high PPCS at follow-up with an area under the curve (AUC) of 0.86 (95% CI 0.80–0.92). IPA identified three significant networks associated with PPCS: (a) cardiovascular and neurological disease, organismal injury, and abnormalities (score = 45; 28 focus molecules); (b) connective tissue disorders, inflammatory disease, and organismal injury and abnormalities (score = 41; 26 focus molecules); and (c) connective tissue development and function, embryonic development, and organismal development (score = 41; 26 focus molecules).

**Conclusion:**

This study suggests the possibility to utilize novel biomarkers discovered by high throughput proteomic analysis to predict high PPCS. Future research should further develop precision of unique biomarker profiles and prolonged symptomatology in adolescents’ post-concussion.

## Introduction

One in five adolescents will sustain a concussion and may experience persisting post-concussion symptoms (PPCS), lasting longer than three months post-injury. PPCS occurring in adolescents following concussion and can significantly disrupt an adolescent’s ability to return to normal daily activities such as academic engagement and social participation (1). Given these impacts, adolescents may experience greater disruptions in academic achievement, emotional development, and overall well-being during a crucial period of cognitive and social maturation (2). However, current methods to predict PPCS have a limited ability to accurately identify specific biological factors that may contribute to prolonged recovery (3, 4). In previous studies of adolescents and adults, predicting PPCS through fluid biomarkers has been limited to examining several common biomarkers, including primarily glial fibrillary acid protein (GFAP), neurofilament light chain (NFL), or tau to PPCS or global recovery, suggesting that these biomarkers have limited prognostic value in predicting traumatic brain injury (TBI) associated recovery (5).

To address these methodological limitations, we undertook a non-hypothesis-based approach to identify novel proteins related to PPCS using Olink Explore to examine 5,400 proteins. We used ingenuity pathway analysis (IPA) to develop hypotheses related to how protein networks were associated with PPCS and to determine protein interaction and proteomic pathways underlying persisting symptoms. Although our biomarker approach was non-hypothesized, we were guided by an overall hypothesis that combinations of proteins would distinguish high PPCS 3 months post-injury with greater sensitivity and specificity than previously examined proteins.

## Methods

The Concussion Assessment, Research, and Education for Kids (CARE4Kids) study (U54 NS121688) is a longitudinal, multi-site investigation aimed at identifying and clinically validating endotypes (i.e., distinct constellations of biological markers that predict outcomes) of PPCS in children and adolescents aged 11 to 17.99 years (6). Participants in our study were recruited from participating outpatient clinics between 2021 to 2024. The study was approved by the central IRB for the data coordinating center, and all participants/parents provided signed informed assent/consent.

### Post-concussion symptom evaluation

PPCS severity was quantified 7–35 days after injury (baseline) and again at 3 months after injury (follow-up) using the Post-Concussion Symptom Inventory, 2nd Edition (PCSI-2). A Retrospective Adjusted Post-Injury Difference (RAPID) score on the PCSI-2 was used that removes the adolescent’s self-reported pre-injury symptoms from their current post-injury symptoms (7). The PCSI-2 is a validated self-report tool covering 20 common post-concussion symptoms, and symptoms are rated on a 7-point ordinal scale, ranging from *none* (0) to *severe* (6). The RAPID score derived from the PCSI-2 responses provides a total overall score with strong psychometric properties (7). This study further defined individuals in the highest 25% post-concussion symptom severity at follow-up as the PPCS-high group and those in the lowest 25% as the PPCS-low group.

### Inclusion and exclusion criteria

For inclusion in the study, participants had to be between the ages of 11 and 17.99 years diagnosed with a concussion by a healthcare provider in accordance with the Concussion in Sport Group (CISG) criteria. Participants also had to be proficient in English, although parental surveys and forms were adapted to accommodate Spanish-speaking parents. Additionally, participants needed to be between 5 to 35 days of post injury at the time of the initial post-injury evaluation (baseline); however, the earliest timepoint that was collected was 7 days post-injury.

Exclusion criteria included significant neurological disorders such as epilepsy, an active severe psychiatric illness or substance use disorder, or severe autism or developmental delays. Adolescents who had sustained concussion within the previous 3 months, or who were still experiencing persisting symptoms from any prior concussion were also not eligible for participation. Individuals with a history of moderate or severe TBI were excluded. However, individuals with a history of migraines, attention deficit hyperactivity disorder (ADHD), or common psychiatric conditions like anxiety or depression were eligible.

### Laboratory analyses for blood-based biomarkers

We examined links of the established acute fluid biomarkers at baseline, GFAP, NFL, ubiquitin carboxy hydrolase-L1 (UCH-L1), tau, and phosphorylated tau (p-tau) 181, to PPCS in adolescents at follow-up using Simoa technology. We included a comparison of protein expression levels from samples collected within 7–20 days and 21–35 days to examine the impacts of time on these biomarkers. In addition, we utilized a non-hypothesis-based approach with proteomics and the Olink technology to assess protein differences between PPCS-high and PPCS-low groups.

GFAP, NFL, UCH-L1, tau, and p-tau181 were assayed using the Quanterix Simoa® Neuro 4-Plex A Advantage Kit (Lot #504046). Phosphorylated tau at threonine 181 (p-tau181) was measured separately using the Simoa® p-Tau-181 Advantage V2.1 Kit (Lot #504313). Simoa (Single Molecule Array) technology, developed by Quanterix®, Inc. (Lexington, MA), enables ultra-sensitive detection of low-abundance proteins by isolating and quantifying individual enzyme complexes within femtoliter-sized wells This digital ELISA approach allows protein concentrations to be measured at sub-femtomolar levels well below the detection threshold of conventional ELISA methods, making it possible to approximate central activity of neuropeptides through peripheral blood samples.

Plasma samples were thawed on wet ice from −80 °C storage, briefly vortexed and spun down to remove precipitate from supernatant, then 110ul were aliquoted onto a pre-assigned well plate for the Simoa HD-X program. These samples were run in duplicate as well as calibrators and controls with an in instrument 4x dilution. Results were produced by the digital immunoassay as well as calibration curve reports. The lower limits of detection for Tau, NFL, GFAP, UCH-L1, and p-Tau-181 were, 0.024 pg./mL, 0.104 pg./mL, 0.221 pg./mL, 1.74 pg./mL, and 0.724 pg./mL, respectively.

### Novel biomarkers

Olink technology is a high-throughput proteomics platform that uses Proximity Extension Assay (PEA) technology to enable precise, multiplex protein biomarker analysis. By employing dual antibody binding and DNA-based detection, it offers high specificity and sensitivity, even in complex biological samples like plasma and serum. Using plasma, the samples were aliquoted to a pre-assigned well within a plate, which was then transferred to one sample source plate using a F. A. S. T.® instrument and samples were diluted using the SPTlabtech Dragonfly® instrument. The immune-reaction or incubation was next, when the High multiplexed PEA™ probes, which were matched pairs of antibodies with unique DNA oligonucleotides, bind to their respective proteins in the samples. This incubation period was between 16–24 h with all of the cohort maintaining the same incubation time for consistency; 18-h incubation was used for this study. On the start of day 2, following appropriate incubation time, the oligonucleotides that were brought into proximity hybridize and are extended using a DNA polymerase. The piece of DNA barcode that was created was then amplified by a Polymerase Chain Reaction (PCR). Unique sample indexes were added to every sample using the F. A. S. T.® to allow pooling of the DNA amplicons for all samples. The final DNA amplicons in the Olink Explore HT libraries include specific barcode sequences for each assay, sample specific indexes, and required sequences for Illumina sequencing (P5 and P7 Adapters and Sequencing Primer Binding Site Rd1SP). Following the PCR procedure, PCR pooling was conducted using the F. A. S. T.® instrument which pools samples from the same dilution block together, resulting in one pool per dilution block, each containing 192 samples/controls. Once complete, the library purification and quality control were performed, using AMPure® XP Magnetic Beads to purify and assess the samples using the Agilent® Tapestation 4,200 instrumentation and High Sensitivity D1000 Tapestation kits to determine quality of samples. The samples were then pooled into the final Explore HT library and sent for sequencing by NGS using Illumina platform. The relative concentration of each biomarker, based on matched counts (the number of reads for each specific combination of sample and assay), was calculated using the Olink NPX Explore HT software.

### Statistical analyses

To evaluate baseline patient demographics and control for potential clinical confounders, statistical analyses of clinically related data were conducted using R (version 4.4.0). Independent t-tests were implemented for continuous variables, and χ^2^ for categorical data. Key clinical covariates, including age, sex, and body mass index (BMI), were strictly balanced and matched across the experimental cohorts during the study design phase. Statistical testing verified that no significant baseline differences existed for these demographic variables between the groups prior to downstream proteomic modeling, eliminating their variance and rendering their inclusion as redundant modeling parameters unnecessary. Established and novel biomarkers measured at the sub-acute time point were compared between cohorts in the highest and lowest quartiles of post-concussion symptom severity 3 months after injury (follow-up).

To develop a biomarker panel from Olink, first biomarker analyses were completed using the Olink Proximity Extension platform within the cohort. The measured raw protein expression levels were normalized using the intensity normalization method to yield Normalized Protein eXpression (NPX) values using a multivariable model. The normalized expression data were then assessed for normality using the Shapiro–Wilk test and were found to be non-normally distributed; therefore, non-parametric tests were used. The Wilcoxon Rank Sum Test (OlinkAnalyze in R) was used to determine whether distributions differed between PPCS-high and PPCS- groups. The Benjamini-Hochberg Procedure False Discovery Rate (FDR) method was used to account for multiple testing.

Protein expression data for overlapping proteins with matched sample IDs were extracted and merged based on unique sample identifiers. To resolve the high-dimensional nature of the dataset (5,400 + proteins) while prioritizing clinical interpretability and avoiding automated black box machine learning selection algorithms, a hybrid, biology-driven dimensionality reduction workflow was implemented to focus the feature space down to a manageable pool of approximately 10 candidate biomarkers. This pre-filtering step combined strict statistical thresholds alongside robust biological priors. First, statistical pre-filtering was implemented to identify highly related univariate proteins to the dataset. Proteins were required to meet stringent univariable thresholds of an unadjusted *p*-value < 0.003 and an absolute log2FoldChange > 0.20. Afterwards, biological pre-filtering was used to identify proteins with high clinical relevance. Ingenuity Pathway Analysis (IPA; QIAGEN Inc., https://digitalinsights.qiagen.com/IPA) was integrated alongside established literature priors from our group to identify pathways with verified pathological relevance. Altered proteins meeting an unadjusted *p*-value cutoff (<0.05) were uploaded to IPA for Core Analysis to determine enriched canonical pathways and map functional interaction networks. These biological relationships were graphically represented with nodes and lines, supported by at least one canonical reference in the QIAGEN knowledge base. Only human orthologs of genes were considered, selecting for proteins that demonstrated coherent biological mechanisms.

Following this pre-filtering dimensionality reduction stage, the focused pool of approximately 10 candidate proteins was evaluated using a traditional statistical modeling framework rather than an automated machine learning approach. To identify the most clinically applicable, reproducible, and parsimonious biomarker panel, an exhaustive best-subsets regression strategy was executed. Standard logistic regression models were fit to evaluate all possible multivariable configurations of the candidate proteins (2^10^–1 = 1,023 total unique models), utilizing protein expression profiles as independent predictors and group status (PPCS-high vs. PPCS-low) as the binary outcome. This exhaustive statistical approach guaranteed that unique, niche multivariable combinations were evaluated without running the risk of overfitting models to background noise.

Receiver operating characteristic (ROC) and area under the curve (AUC) analysis was performed on the cross-validated probability predictions, and bootstrapping with 200 resamples was used to calculate the 95% confidence intervals for the cross-validated AUC scores. The optimal cutoffs to differentiate PPCS-high and PPCS-low groups were determined using the “top-left” method to calculate sensitivity, specificity, positive predictive value (PPV, precision), and negative predictive value (NPV). Final regression coefficients and deployment equations were extracted from the complete models fit across the entire dataset.

## Results

### Participant characteristics

**Figure 1:**
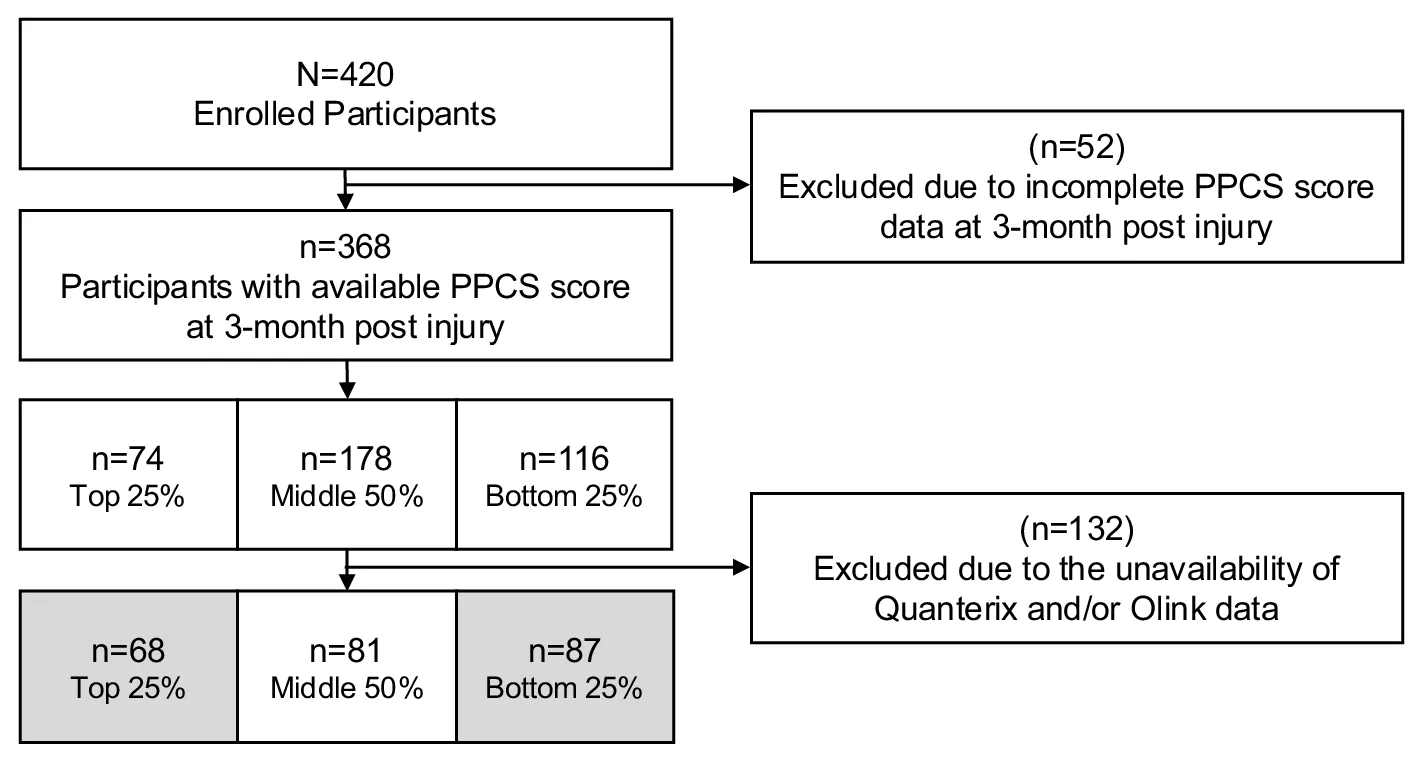
Diagram of study inclusion and exclusion.

A total of 155 participants with both blood biomarker data and 3-month symptom data were included in this study (Figure 1). The demographic characteristics of the cohort are summarized in Table 1. While baseline clinical traits such as age and body mass index (BMI) were strictly balanced during cohort selection to eliminate them as potential confounders, certain demographic and injury characteristics varied between the groups. The cohort included more females in the PPCS-high group, 67.6% vs. 32.4% (*p* < 0.01). The high PPCS group also had a greater number of days between injury and the sub-acute data collection (*p* < 0.001), as well as lower rates of sports concussion as the cause of injury (*p* = 0.002).

**Table 1:** Demographics of cohort.

Characteristic	PPCS-high (*n* = 68)	PPCS-low (*n* = 87)	Total (*n* = 155)	*p*-value
Sex, *n* (%)				**<0.001**
Male	22 (32.4)	55 (63.2)	77 (49.7)	
Female	46 (67.6)	32 (36.8)	78 (50.3)	
Ethnicity, *n* (%)				0.751
Not Hispanic/Latino	49 (72.1)	63 (72.4)	112 (72.3)	
Hispanic/Latino	15 (22.1)	21 (24.1)	36 (23.2)	
Unknown	4 (5.9)	3 (3.4)	7 (4.5)	
Race, *n* (%)				0.590
White	39 (57.4)	53 (60.9)	92 (59.4)	
Black or African American	11 (16.2)	13 (14.9)	24 (15.5)	
Asian	2 (2.9)	4 (4.6)	6 (3.9)	
More than 1 race	11 (16.2)	7 (8.0)	18 (11.6)	
American Indian	0 (0.0)	1 (1.1)	1 (0.6)	
Unknown	5 (7.4)	9 (10.3)	14 (9.0)	
Age, mean ± SD	15.1 ± 1.8	14.8 ± 1.8	15 ± 1.7	0.237
BMI, mean ± SD	21.9 ± 4.6 (*n* = 52)	21.5 ± 4.3 (*n* = 64)	21.7 ± 4.4	0.565
Days between injury and follow-up, mean ± SD	24.9 ± 7.6	18.7 ± 7.5	21.5 ± 8.2	<0.001
RAPID overall score at follow-up, mean ± SD	30.3 ± 14.2	0.0 ± 0.0	13.6 ± 17.8	<0.001
Injury mechanism, *n* (%)				**0.002**
Sports	26 (38.2)	54 (62.1)	80 (51.6)	
Motor vehicle	12 (17.6)	4 (4.6)	16 (10.3)	
Fall	10 (14.7)	7 (8.0)	17 (11.0)	
Assault	3 (4.4)	1 (1.1)	4 (2.6)	
Other	3 (4.4)	1 (1.1)	4 (2.6)	
Missing	6 (8.8)	14 (16.1)	20 (12.9)	

BMI, Body mass index; baseline: 7–35 day post-injury evaluation; follow-up: 3-month post-injury evaluation RAPID: Retrospective Adjusted Post-Injury Difference.

### Fluid biomarkers related to PPCS

Table 2 provides the medians and interquartile ranges for the established fluid biomarkers collected at the subacute appointment. There were no significant differences in biomarkers between the two groups (PPCS-high and PPCS-low) based on FDR-corrected *p*-values. We also compared levels of these biomarkers between the two groups during the sub-acute period (7–20 days) and the extended period (21–35 days), and there were no significant differences (*p*’s > 0.20).

**Table 2:** Candidate brain biomarker levels at baseline between PPCS-high and PPCS-low groups at follow-up.

Biomarker	PPCS-high	PPCS-low	*p*-value
	Median (Q1, Q3)	Median (Q1, Q3)	
GFAP	80.1 (63.1, 96.8)	79.8 (63.2, 94.1)	0.879
NFL	3.9 (2.9, 5.2)	3.7 (2.9, 5.0)	0.846
Tau	4.9 (3.6, 6.6)	4.7 (3.5, 6.1)	0.352
P-tau	15.5 (13.2, 20.2)	15.8 (12.6, 19.9)	0.813
UCH-L1	15.3 (9.3, 33.1)	15.3 (8.8, 21.2)	0.423

Q1, first quartile; Q3, third quartile.

Table 3 shows the top 20 proteins at baseline that most distinguish PPCS-high from PPCS-low at follow-up within the cohort, along with their primary functions. These proteins were among 296 that passed the p-value cutoff, with 158 down-regulated and 138 up-regulated. They display a range of fold-change differences between the PPCS-high and PPCS-low groups, from −1.68 to 1.37. The difference column reflects the magnitude of the difference per-protein in the group comparisons.

**Table 3:** Top 20 proteins at the subacute appointment that were different between PPCS-high and PPCS-low groups based on data at 3-months.

Protein	Primary function	log2FoldChange	Log2 average PPCS-low	Log2 average PPCS-high	*p*-value
Persephin (PSPN)	Promotes survival of neurons	−0.7828	0.1584	−0.6244	4.68E-05
Oviductal Glycoprotein1 (OVGP1)	Follicular development	1.3681	0.1386	1.5067	0.00014
T-Cell surface glycoprotein (CD4)	Antigen presenting cell	−0.3369	0.0895	−0.2474	0.000407
Testis expressed 101 (TEX101)	Sperm activity	−0.7208	0.6331	−0.0877	0.000421
Tripartite motif containing 39 (TRIM39)	Major histocompatibility complex	0.7264	−0.3617	0.3647	0.000669
Sclerostin (SOST)	Bone morphogenic	−0.3147	−0.0555	−0.3702	0.000767
Transcription factor Dp family member 3 (TFDP3)	DNA transcription factor	−0.4074	0.0749	−0.3324	0.00092
TNF receptor superfamily member 9 (TNFRSF9)	Development of T-cells	−0.6644	0.1047	−0.5597	0.000957
Leptin (LEP)	Regulates energy homeostasis	0.9651	−0.8754	0.0898	0.00106
Prune homolog 2 with BCH domain (PRUNE2)	Apoptosis and synaptic function	−0.4706	0.0557	−0.4149	0.00106
Trimethyl guanosine synthase 1 (TGS1)	Trimethylguanosine synthase activity	−0.3578	0.1638	−0.1940	0.00125
Epithelial cell adhesion molecule (ECAM)	Carcinoma-associated antigen	0.4942	−0.2753	0.2190	0.001348
Chordin Like 2 (CHRDL2)	Transforming growth factor beta	0.2634	−0.2242	0.0392	0.001471
Glypican 1 (GPC1)	Cell division and growth regulation	−0.5575	0.0885	−0.4690	0.001584
Hydroxyacyl-CoA Dehydrogenase (HADH)	Mitochondrial activity	0.8441	−0.7752	0.0689	0.001584
Collagen Type IX Alpha 1 Chain (COL9A1)	Collagen promotion	−1.2560	0.6636	−0.5924	0.001624
Tetraspanin 1 (TSPAN1)	Cell development, activation, growth	0.2146	0.0275	0.2422	0.001706
Sperm equatorial segment protein 1 (SPESP1)	Spermatogenesis	−0.3615	0.1638	−0.1977	0.00177
Acrosomal Vesicle Protein 1 (ACRV1)	Spermatogenesis	−0.2895	0.2819	−0.0076	0.001976
Insulin like growth factor binding protein Like 1 (IGFBP1)	Stimulate the growth-promoting effects	0.2256	−0.1451	0.0805	0.002425

### Combined predictors of high PPCS

**Figure 2:**
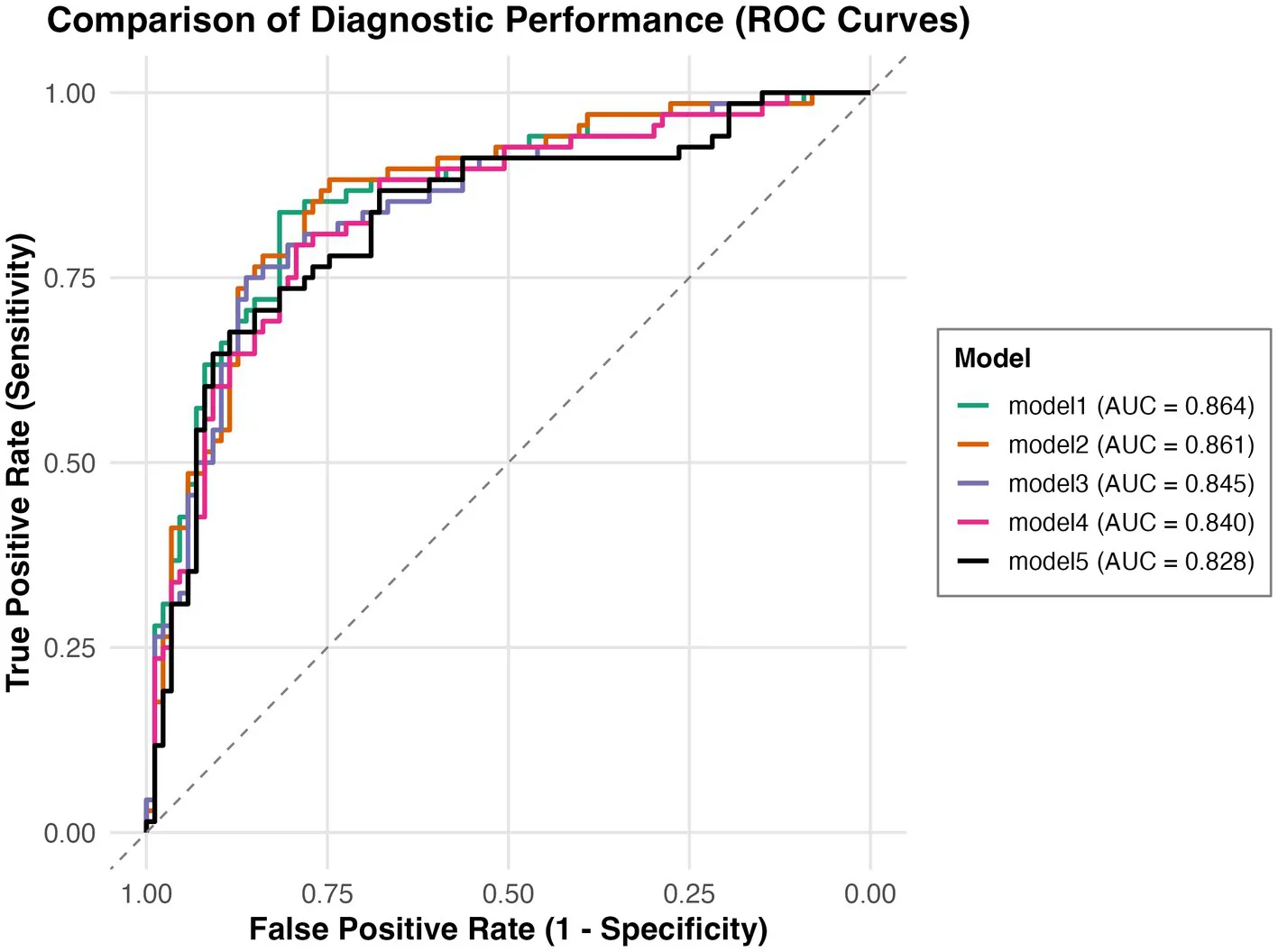
Comparison of receiver operator characteristic (ROC) curves of the top 5 protein combinations.

We examined combinations of proteins at baseline that best distinguished PPCS-high from PPCS-low participants at follow-up using AUC analyses. The top 5 combinations were included based on discriminative ability to prioritize combinations that maximize true positive rates and NPVs (see Table 4 and Figure 2). Of the 20 top-differentiating proteins, 4 proteins (Oviductal Glycoprotein1 [OVGP1], Testis Expressed 101 [TEX101], Sperm Equatorial Segment Protein 1 [SPESP1], and Acrosomal Vesicle Protein 1 [ACRV1]), were predominately related to sex-specific functions. To address this sex-related biological variation, we performed AUCs excluding proteins known to be related to sex, including: OVGP1, TEX101, SPESP1 and ACRV1. We also adjusted for sex in the multivariable model to account for other protein expressions indirectly influenced by sex and developmental stage in this adolescent population.

**Table 4:** Area under the curve (AUC) analysis of models distinguishing between PPCS-high and PPCS-low groups.

Model	Proteins	AUC	95% CI low	95% CI high	Sensitivity	Specificity	PPV	NPV
model1	Sex + TRIM39 + SOST + TFDP3 + TNFRSF9 + LEP + PRUNE2 + TGS1 + EPCAM	0.864	0.800	0.919	0.838	0.816	0.781	0.866
model2	Sex + TRIM39 + SOST + TFDP3 + TNFRSF9 + LEP + PRUNE2 + EPCAM	0.861	0.793	0.921	0.838	0.782	0.750	0.861
model3	Sex + PSPN + CD4 + TRIM39 + SOST + TFDP3 + EPCAM	0.845	0.775	0.909	0.794	0.805	0.761	0.833
model4	Sex + CD4 + TRIM39 + SOST + TFDP3 + EPCAM	0.840	0.773	0.909	0.794	0.793	0.750	0.831
model5	Sex + CD4 + TRIM39 + TFDP3 + EPCAM	0.828	0.755	0.892	0.735	0.816	0.758	0.798

PPV, positive predictive value; NPV, negative predictive value.

To rigorously evaluate the internal validity and generalizability of our final configurations, we executed a stratified 10-fold cross-validation framework at the conclusion of our modeling pipeline. Operating as an independent verification layer rather than an iterative optimization loop, this cross-validation generated out-of-fold probability predictions to verify model stability and safeguard against overfitting. The cross-validated AUC scores closely tracked the full-dataset AUC estimates, with all cross-validated AUCs falling within the corresponding 95% confidence intervals of the original models, indicating excellent model stability. Complete out-of-fold performance metrics are provided in Supplementary Figure S1; Supplementary Table S1.

### Enriched pathways and networks of proteins related to PPCS

**Figure 3:**
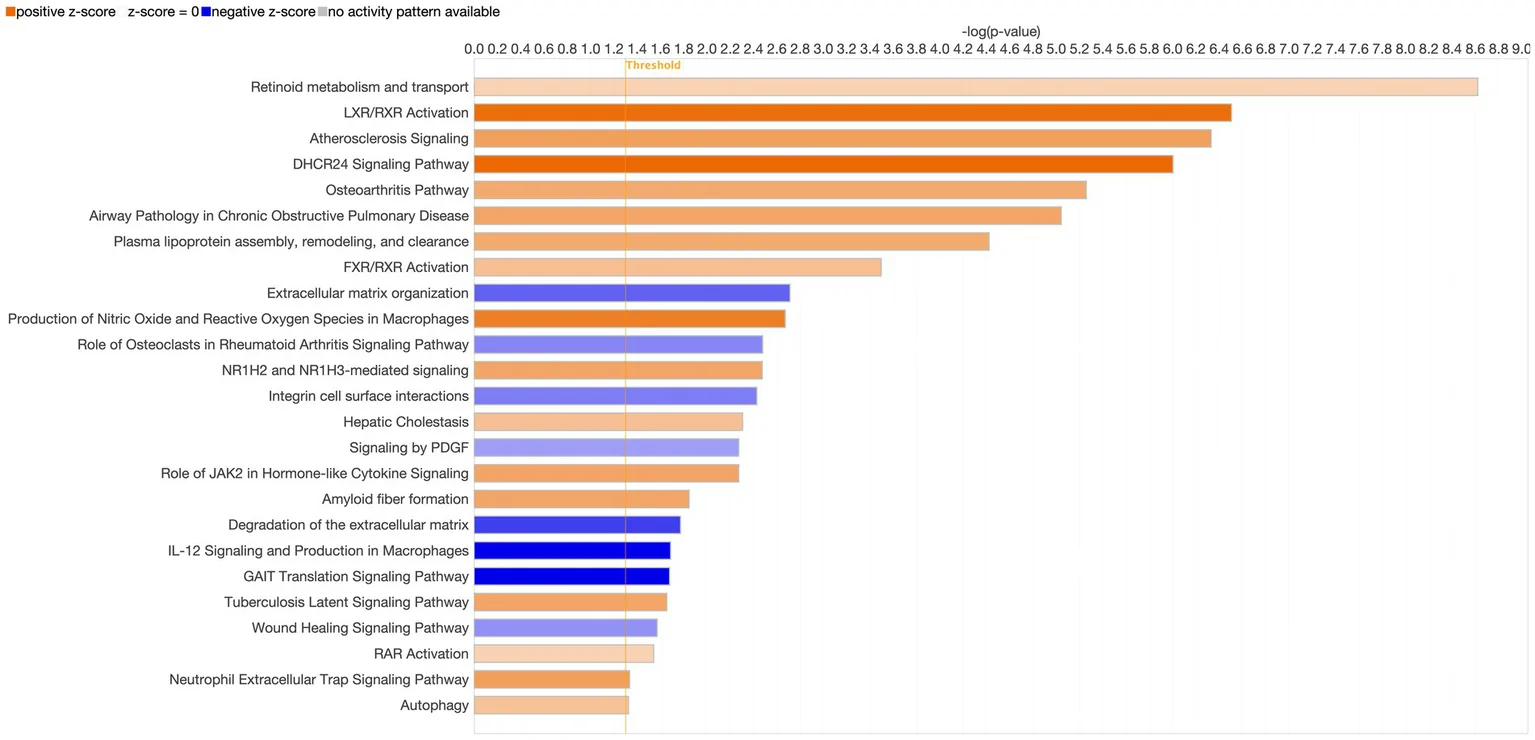
Canonical pathways enriched with proteins dysregulated in PPCS.

IPA provided some initial insights into canonical pathways in which the proteins were enriched (Figure 3). Specifically, LXR/RXR activation and PHCR24 signaling pathways as well as amyloid fiber formation pathways were predicted to be activated in the PPCS-high group while IL-12 signaling and production in macrophages pathways were inhibited based on the observed protein level differences.

**Figure 4:**
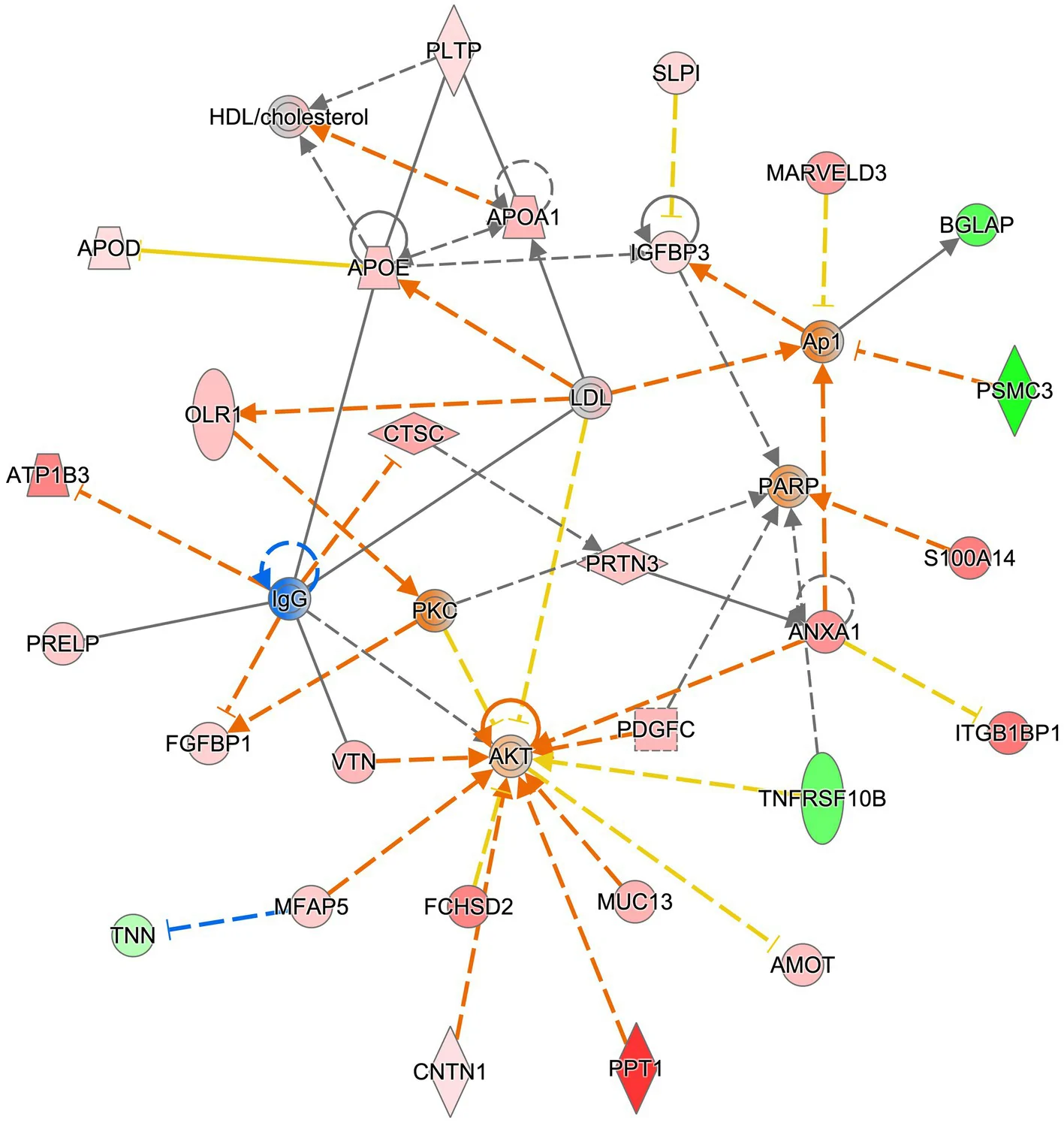
Network of cardiovascular disease, neurological disease, organismal injury and abnormalities. Node color and intensity reflect the observed degree of gene expression dysregulation, with red (upregulation) and green (downregulation). Predicted activation states of genes within the network are shown in orange (activated) and blue (inhibited), with relationships represented by solid lines (direct) or dashed lines (indirect). Lines in orange, blue, yellow, and gray indicate relationships between nodes that lead to activation, inhibition, inconsistent findings, and an unexpected effect, respectively.

**Figure 5:**
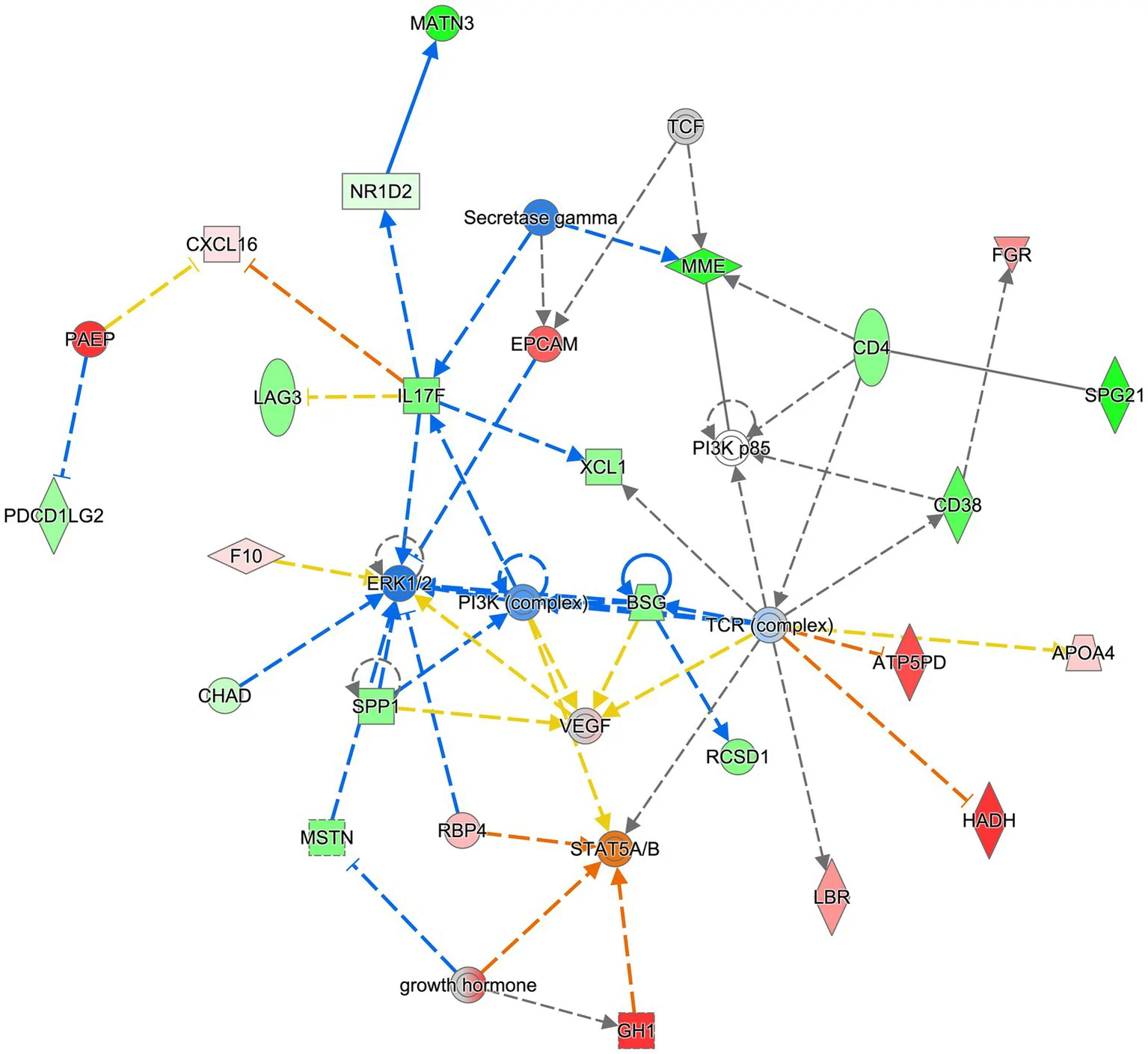
Network of connective tissue disorders, inflammatory disease, organismal injury and abnormalities. Node color and intensity reflect the observed degree of gene expression dysregulation, with red (upregulation) and green (downregulation). Predicted activation states of genes within the network are shown in orange (activated) and blue (inhibited), with relationships represented by solid lines (direct) or dashed lines (indirect). Lines in orange, blue, yellow, and gray indicate relationships between nodes that lead to activation, inhibition, inconsistent findings, and an unexpected effect, respectively.

**Figure 6:**
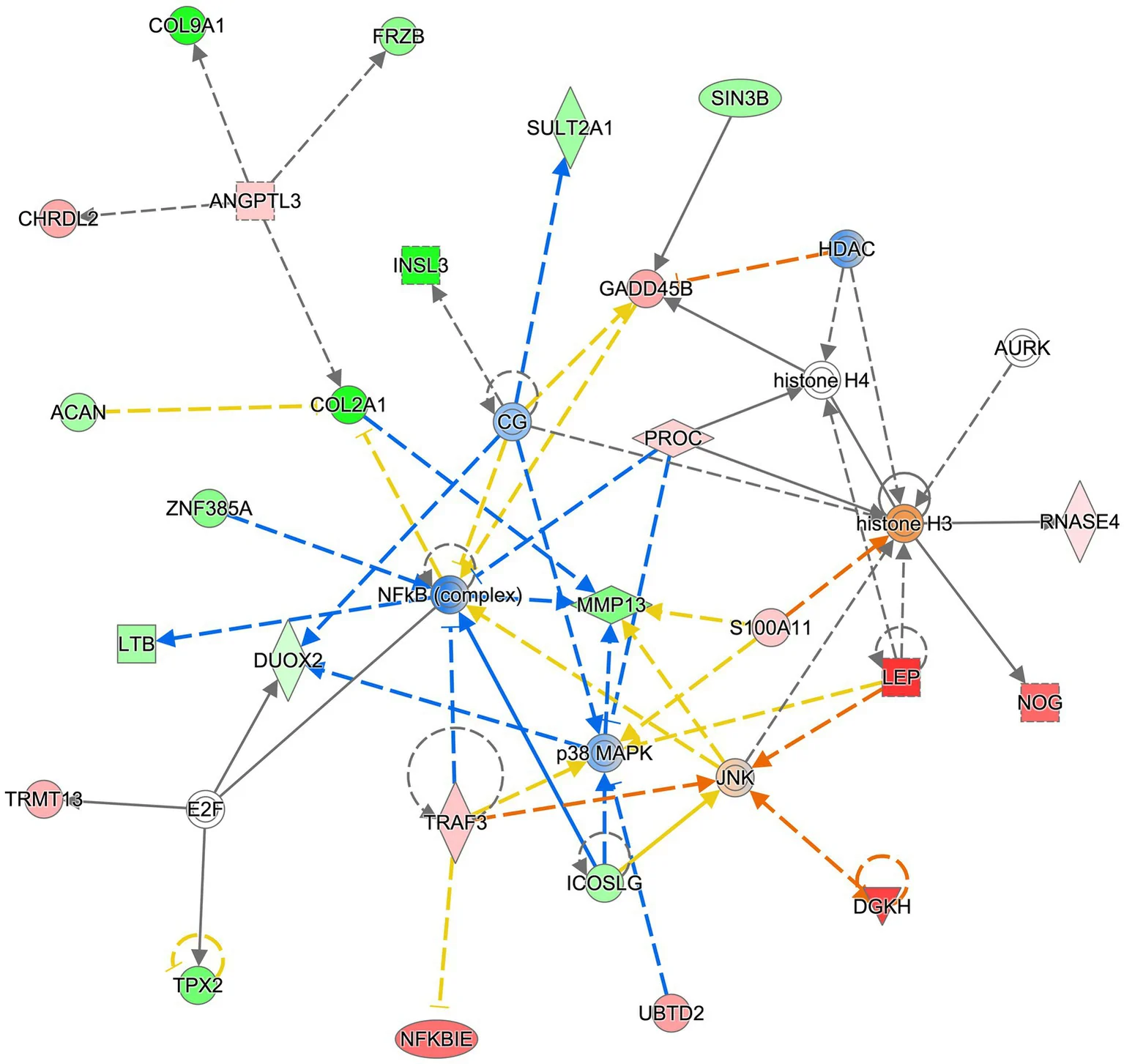
Network of connective tissue development and function, embryonic development, organismal development. Node color and intensity reflect the observed degree of gene expression dysregulation, with red (upregulation) and green (downregulation). Predicted activation states of genes within the network are shown in orange (activated) and blue (inhibited), with relationships represented by solid lines (direct) or dashed lines (indirect). Lines in orange, blue, yellow, and gray indicate relationships between nodes that lead to activation, inhibition, inconsistent findings, and an unexpected effect, respectively.

Lastly, we used IPA to determine the top 3 performing networks of the proteins (Figures 4–6). Pathways included: a. Cardiovascular disease (CVD), neurological disease, organismal injury and abnormalities (−log10p = 45; number of focus molecules = 28); b. Connective tissue disorders, inflammatory disease, organismal injury and abnormalities (−log10p = 41; number of focus molecules = 26); and c. Connective tissue development and function, embryonic development, organismal development (−log10p = 41; number of focus molecules = 26).

## Discussion

Here we report initial exploratory work to suggest potential novel proteins, protein pathways, and networks related to high PPCS at 3 months after concussion. These findings are related to protein combinations and patterns, not single proteins, as no proteins remained significant after adjusting for multiple comparisons. One notably differentially expressed protein was persephin (PSPN), a part of the glial cell line-derived neurotrophic factor (GDNF), which was the most significantly down-regulated protein in the PPCS-high group. PSPN has potent trophic effects on adult sensory neurons after nerve injury (8, 9). In previous studies of hypoxia-based brain injury, it was reported that mice lacking PSPN were more sensitive to ischemic brain damage, but the damage could be recovered by an injection of PSPN (10). PSPN promoted the survival of both motor and dopamine neurons, both *in vitro* and *in vivo* (11, 12). A recent study of mesenchymal stem cells secreting PSPN showed that PSPN regulates the proliferation and differentiation of neural stem cells *in vitro*; however, it did not show improvements in outcome after hypoxic brain injury in mice (13). Our results showed that a low level of PSPN at baseline was associated with high PPCS at follow-up, indicating the protein may play a beneficial role in recovery from brain injury.

Another downregulated protein associated with high PPCS at follow-up is glypican-1 (GPC1), a protein critical to neuronal differentiation and growth (14). In an *in vitro* model, suppression of GPC1 resulted in increased phosphorylation at tau sites 181 and 217, and that when overexpressed, phosphorylation of tau 217 decreased (15). Since tau increases are considered standard in TBI, reflecting axonal disruption, and studies show that hyperphosphorylation relates to both short and long-term neurological implications (16), this finding provides some initial insights into the nature of these links, within a young cohort. Although p-tau-181 level did not differ significantly between groups in this study, the observed difference in GPC1, an upstream regulator of tau phosphorylation, raises an avenue for further investigation. Given the role of post-translational modification and other regulatory mechanisms in human biology, GPC-1 could affect signaling pathways relevant to tau phosphorylation but may not be reflected solely by p-tau-181 level.

We observed an approximately two-fold greater leptin (LEP) level in the high PPCS group, with a strong association with high PPCS when combined with 6–7 other dysregulated proteins (see models 1 and 2). Leptin has been previously suggested as a prognostic factor for poor 6-month clinical outcomes (mortality and Glasgow Outcome Scale of 1–3) in children with moderate–severe TBI (17). Leptin regulates several cytokine secretion patterns and participates in the inflammatory process based on literature showing that its production is regulated by IL-1, IL-6, and TNF-alpha, as well as regulating production of CRP (18, 19). In addition, TBI induces central nervous system disruption and inflammation, which can lead to compromised blood–brain barrier, resulting in upregulation of leptin (20). Leptin pathway is associated with skeletal fragility after TBI, one of the long-term health consequences (20). However, some models reported leptin’s protective effect in cerebral ischemic injury (21, 22), suggesting the relationship between leptin and brain injury remains complex.

The predominant network (Figure 4) identified is highly related to AKT (serine/threonine protein kinases) activation, and related protein activities. Following a TBI, activation of AKT/mTOR signaling pathway plays a role in the formation of new axons and synapses, which facilitates the recovery of sensorimotor functions through cortical remapping (23). A study reported that AKT phosphorylation increases after TBI in mice and is involved in cell survival (24). The network also identifies that PDGFC (Platelet-Derived Growth Factor C) activation will promote AKT signaling pathways. The activation of PDGFC also triggers blood–brain barrier (BBB) dysfunction and inflammation signaling (25), which may contribute to persisting symptoms after injury. Within this first network, in the context of decreased cellular growth, there is an increase in proteins related to immune regulation. Specifically, a central node is immunoglobulin (IgG), a key regulator of the immune system, allowing for protection from antigens. This finding in the context of our observation of down-regulated activity within the PPCS-high group with dysregulation of other immune proteins (including TNF Receptor Superfamily Member 9 [TNFRSF9], T-Cell Surface Glycoprotein [CD4], and others) as well as Annexin-A1 Tripeptide (ANXA1sp), which is linked to mitigating neuronal loss post-TBI via immune regulation (26). Further, within network 3 (Figure 6), we also observe nuclear-factor kappa (NFKB) be a downregulated node, suggesting that PPCS risks relate to immune dysregulation. Previous studies link immune regulation as a key mediator in TBI recovery (27), and a systematic review of inflammatory cytokines and TBI prognosis reported that higher levels of MCP-1/CCL2, IL-6, and TNF-*α* are associated with greater PPCS burden, such as higher post-concussion symptom score, duration of symptoms, and days to recovery (28). Synthesizing the concurrent upregulation of leptin with the activation of PDGFC paints a cohesive neurobiological picture of the post-concussive state. It suggests that chronic, low-grade neuroinflammation combined with sustained BBB permeability acts as a core pathophysiological engine, driving the persistence of symptoms long past the expected recovery window.

In this analysis, we observed a higher percentage of females in PPCS-high and males in PPCS-low groups, and in our top performing proteins there were some sex-specific proteins significantly associated with high PPCS. Therefore, some of the observed protein differences may reflect sex-related biological variation rather than being solely attributable to PPCS. While these findings highlight proteins of potential interest, the current analyses cannot fully disentangle sex effects from PPCS-related changes. To prevent overfitting based on the observed findings, we proposed a model that combines dysregulated proteins, excluding sex-specific proteins, while maintaining a comparable AUC. Furthermore, because clinical traits like BMI were evenly balanced across our groups by design, our modeling resources could be strictly dedicated to statistically adjusting for these un-balanced variances in sex. However, the sex-based difference in PPCS has been reported in the literature and thus may be important to consider. Previous studies show a greater impact of PPCS in females, such as higher prevalence of PPCS and greater symptom severity as well as longer recovery time (29–31). This finding has been partially explained by psychological and emotional factors, such as anxiety sensitivity (32), but it remains unknown if persisting symptoms in females are related to biological sex-based differences. To address this gap, future studies with sex-balanced cohorts or stratified analyses are warranted to clarify the contributions of sex to high PPCS.

Candidate proteins associated with brain injuries, including GFAP, NFL, tau, p-tau, and UCH-L1 were tested. However, none of them showed significant differences between the two PPCS groups subacutely. Further, as a sensitivity analysis, we examined proteins in samples available from 7 to 20 days, versus beyond 21 days to examine more early and later collected samples, a division made based on the median days from injury to blood draw, showing no significant difference. Previously these biomarkers, except p-tau, have been shown to mostly peak within a few hours to a few days after injuries and decline thereafter (33). Given the sensitivity to the early time window, the study’s blood-drawing time frame, which was within 7–35 days of injury, may not reflect these changes. However, this 7- to 35-day window is clinically relevant, as it aligns with the subacute phase when adolescents typically present to specialized outpatient concussion clinics with lingering symptoms (34). A biomarker panel that is robust in this specific timeframe would provide vastly more clinical utility for outpatient prognostic management than acute markers, which are currently largely restricted to emergency department settings to rule out the need for neuroimaging (35). Moreover, the established markers utilized for this study are most closely related to severity of brain injury rather than persistence of symptoms, which is likely governed by a variety of underlying biomarker changes. Although all participants sustained a concussion, only a subset reported high PPCS, suggesting the need to identify new markers as reliable indicators of PPCS. Ultimately, validating these biological markers—particularly the identified immune and connective tissue pathways—could provide clinicians with a much-needed objective tool. A reliable fluid biomarker profile would allow healthcare providers to distinguish structurally or metabolically driven prolonged recovery from cases with overlapping symptomatology that might require primary behavioral intervention.

Although the findings of this study offer exploratory insights and help generate hypotheses for further research, several limitations should be acknowledged. These findings were based on comparisons of the most extreme symptoms at both the highest and lowest levels; thus, caution is necessary in their interpretation. Future studies may benefit from examining protein expression levels in relation to continuous symptom severity, as well as other relevant possible clinical variables that could be examined in a continuous manner. Also, we were not able to examine important clinical factors including previous TBIs or mental health. The relatively small sample size requires validation to inform the next steps and translate the findings, both with this cohort in the ongoing CARE4Kids study and through external validation in independent cohorts. Although the proteins that passed the cutoff *p*-value significance level revealed trends in biological pathways related to PPCS, individual proteins did not survive after FDR correction. Furthermore, the sex imbalance between groups may have influenced protein expression patterns, reinforcing the need for future studies with a larger sample size and balanced sex distribution when plausible. Even after statistically adjusting for the effect of sex, the confounding from sex may remain. In addition, although a sensitivity analysis of time since injury and biomarker levels showed no differences between the groups, the confounding effects of time since injury on biomarker levels may not yet be fully addressed, as a greater sample size will allow for this in future analyses. Methodologically, the Olink platform does not capture post-translational modifications and provides a normalized standard value rather than absolute quantification; validation using orthogonal methods such as ELISA and other protein assays will be essential to confirm these findings.

## Data Availability

The proteomics dataset generated in this study has been deposited in the PRIDE repository through the ProteomeXchange Consortium under accession number PAD000053. The dataset is available at https://www.ebi.ac.uk/pride/archive/projects/PAD000053. De-identified clinical data supporting the findings of this study are available from the corresponding author upon reasonable request, subject to institutional review board approval and applicable ethical and data-sharing requirements.
