# Supplementary material for: Exploratory proteomic biomarker combinations associated with post-concussion symptoms following a concussion in adolescents
Source: Front Neurol. 2026 Aug 14;17:1872460. doi: 10.3389/fneur.2026.1872460 (PMC13523134; doi:10.3389/fneur.2026.1872460)
Supplement: Supplementary file 1 [file Data_Sheet_1.docx]

Supplementary Table 1: 10-fold Cross Validation (CV) of Area under the curve (AUC) models distinguishing between PPCS-high and PPCS-low groups

| Model | Predictors | CV AUC | Sensitivity | Specificity | PPV | NPV |
| --- | --- | --- | --- | --- | --- | --- |
| model1 | Sex + TRIM39 + SOST + TFDP3 + TNFRSF9 + LEP + PRUNE2 + TGS1 + EPCAM | 0.815 | 0.779 | 0.759 | 0.716 | 0.815 |
| model2 | Sex + TRIM39 + SOST + TFDP3 + TNFRSF9 + LEP + PRUNE2 + EPCAM | 0.817 | 0.735 | 0.805 | 0.746 | 0.795 |
| model3 | Sex + PSPN + CD4 + TRIM39 + SOST + TFDP3 + EPCAM | 0.813 | 0.721 | 0.828 | 0.766 | 0.791 |
| model4 | Sex + CD4 + TRIM39 + SOST + TFDP3 + EPCAM | 0.815 | 0.750 | 0.793 | 0.739 | 0.802 |
| model5 | Sex + CD4 + TRIM39 + TFDP3 + EPCAM | 0.795 | 0.662 | 0.862 | 0.789 | 0.765 |

Supplementary Figure 1. Comparison of Receiver Operator Characteristic (ROC) Curves of the Top 5 Protein Combinations After Cross Validation
